# Real-World Impact of Adjuvant Anti-HER2 Treatment on Characteristics and Outcomes of Women With HER2-Positive Metastatic Breast Cancer in the ESME Program

**DOI:** 10.1093/oncolo/oyad137

**Published:** 2023-08-17

**Authors:** Fanny Le Du, Matthieu Carton, Thomas Bachelot, Mahasti Saghatchian, Barbara Pistilli, Etienne Brain, Delphine Loirat, Laurence Vanlemmens, Thomas Vermeulin, George Emile, Anthony Gonçalves, Mony Ung, Marie Robert, Anne Jaffre, Isabelle Desmoulins, Christelle Jouannaud, Lionel Uwer, Jean Marc Ferrero, Marie-Ange Mouret-Reynier, William Jacot, Michaël Chevrot, Suzette Delaloge, Véronique Diéras

**Affiliations:** Department of Medical Oncology, Centre Eugène Marquis, Rennes, France; Department of Biostatistics, Institut Curie, Saint-Cloud, France; Department of Medical Oncology, Centre Léon-Bérard, Lyon, France; Department of Medical Oncology, Hôpital Américain, Paris, France; Department of Cancer Medicine, Gustave Roussy, Villejuif, France; Department of Medical Oncology, Institut Curie/Saint Cloud, Paris, France; Department of Biostatistics, Institut Curie, Saint-Cloud, France; Department of Medical Oncology, Centre Oscar Lambret, Lille, France; Centre Henri Becquerel, Rouen, France; Department of Medical Oncology, Centre François Baclesse, Caen, France; Department of Medical Oncology, Institut Paoli-Calmettes, Marseille, France; Department of Medical Oncology, Institut Claudius Regaud, IUCT-Oncopole, CRCT, Inserm, Toulouse, France; Department of Medical Oncology, Institut de Cancérologie de l’Ouest - René Gauducheau, Saint-Herblain, France; Anne jaffré Department of Medical Information, Institut Bergonié, Bordeaux, France; Department of Medical Oncology, Centre Jean-Francois Leclerc, Dijon, France; Department of Medical Oncology, Institut Godinot, Reims, France; Institut de Cancérologie de Lorraine, Nancy, France; Department of Medical Oncology, Centre Antoine Lacassagne, Nice, France; Department of Medical Oncology, Clermont-Ferrand, France; Department of Medical Oncology, Institut du cancer de Montpellier, Montpellier, France; Health Data and Partnership Department, Unicancer, Paris, France; Department of Cancer Medicine, Gustave Roussy, Villejuif, France; Department of Medical Oncology, Centre Eugène Marquis, Rennes, France

**Keywords:** breast cancer, HER2 positive, anti-HER2-targeted agents, trastuzumab, pertuzumab, de novo metastatic

## Abstract

**Background:**

Although adjuvant cancer treatments increase cure rates, they may induce clonal selection and tumor resistance. Information still lacks as whether (neo)adjuvant anti-HER2 treatments impact the patterns of recurrence and outcomes of HER2-positive (HER2+) metastatic breast cancer (MBC). We aimed to assess this in the large multicenter ESME real-world database.

**Patients and Methods:**

We examined the characteristics and outcomes (overall survival (OS) and progression-free survival under first-line treatment (PFS1)) of HER2+ patients with MBC from the French ESME program with recurrent disease, as a function of the previous receipt of adjuvant trastuzumab. Multivariable analyses used Cox models adjusted for baseline demographic, prognostic factors, adjuvant treatment received, and disease-free interval.

**Results:**

Two thousand one hundred and forty-three patients who entered the ESME cohort between 2008 and 2017 had a recurrent HER2+ MBC. Among them, 56% had received (neo)adjuvant trastuzumab and 2.5% another anti-HER2 in this setting. Patients pre-exposed to trastuzumab were younger, had a lower disease-free interval, more HR-negative disease and more metastatic sites. While the crude median OS appeared inferior in patients exposed to adjuvant trastuzumab, as compared to those who did not (37.2 (95%CI 34.4-40.3) versus 53.5 months (95% CI: 47.6-60.1)), this difference disappeared in the multivariable model (HR = 1.05, 95%CI 0.91-1.22). The same figures were observed for PFS1.

**Conclusions:**

Among patients with relapsed HER2+ MBC, the receipt of adjuvant trastuzumab did not independently predict for worse outcomes when adjusted to other prognostic factors.

## Teaser

There is limited real-world information on the impact of adjuvant and neoadjuvant anti-HER2-targeted treatments on patterns of recurrence and outcomes of patients with HER2+ metastatic breast cancer. This article reports on how anti-HER2 targeted treatment in the early setting impacts overall and progression-free survival under first-line treatment of patients with metastatic HER2+ breast cancer.

**Implications for Practice**

Anti-HER2-targeted therapies have dramatically improved the outcome of patients with HER2+ localized breast cancer. However, it is documented that adjuvant treatments might increase negative clonal selection and tumor resistance at metastatic relapse. In this large ESME registry-based study, adjuvant anti HER2 was associated with more aggressive diseases but did not independently impact survival outcomes of patients with HER2+ relapsed MBC. However, an adjuvant therapy-related shortening of survival (ATRESS) phenomenon cannot be ruled out for patients who experience late relapse.

## Introduction

Amplification of the *HER2* gene used to be associated with an aggressive disease course that has been significantly improved by HER2-targeted therapies. Trastuzumab was introduced 20 years ago and profoundly modified the standard of care in HER2-positive (HER2+) breast cancers, first in the metastatic setting then in the adjuvant setting.^1-4^

Currently, one year of trastuzumab is still the backbone of the adjuvant systemic treatment of all patients with invasive HER2+ early breast cancers.^3,5^ Pertuzumab has also been approved in the early setting as neoadjuvant or adjuvant treatment for patients with early-stage BC with high risk of recurrence (tumor > 2 cm or lymph node involvement) based on the NeoSphere and the Aphinity trials.^6,7^ More recently, trastuzumab emtansine (T-DM1) was approved in the adjuvant setting for patients with residual disease post standard trastuzumab-containing neoadjuvant treatments. The phase III KATHERINE trial which compared T-DM1 to trastuzumab in patients with residual disease after neoadjuvant chemotherapy showed a significant invasive disease-free survival (iDFS) benefit in favor of T-DM1 (HR 0.50 [95%CI 0.39-0.64]; *P* < .001).^8^

It is well known that de novo metastatic HER2+ patients with breast cancer have better outcomes than women with metastatic relapse.^9^ This could be related both to a lead time bias, as well as to an ATRESS (adjuvant therapy-related shortening of survival) phenomenon.^10^ Indeed, cancer treatments may induce clonal selection, as well as tumor resistance and aggressiveness, which could explain a reduced progression-free survival (PFS) at relapse and a significant impact on overall survival (OS).^9,11^

In the Epidemiological Strategy and Medical Economics (ESME) database, up to 60% of the HER2+ MBC population have de novo metastatic cancer in the most recent years. This number has increased over time, probably due to more extensive metastatic work up examinations at diagnosis but also mainly to the major improvements of adjuvant anti-HER2 systemic treatments leading to a considerable relative decrease in relapses.^9^

However, there is still limited real-world information on the impact of adjuvant and neoadjuvant anti-HER2 targeted treatments on patterns of recurrence and outcomes of patients with HER2+ MBC. The purpose of this study was to determine how anti-HER2 targeted treatment in early setting impact OS and progression-free survival under first-line treatment of patients with metastatic HER2+ breast cancer.

## Patients and Methods

### ESME Data Platform and Study Population

In 2014, the 18 French Cancer Centers launched the ESME program to provide real-world data on patients with metastatic breast cancer (MBC). It is an ongoing unique national cohort collecting real-life information built from retrospective data on all consecutive patients treated for MBC in these centers since January 1, 2008.

For the present study, data were collected until the cut-off date of December 31, 2017. Eligible patients were females, aged ≥ 18 years, who initiated their MBC treatment during this period. Collected data include patient demographic characteristics, pathology, outcomes, and treatment patterns.

The ESME research program is managed by Unicancer in accordance with current best practice guidelines and rules.^12,13^ It is supervised by a scientific independent steering committee which approved the present work. This work was authorized by the French data protection authority ([Registration ID 1704113 and authorization N_DE-2013.-117], NCT03275311). Moreover, in compliance with the applicable European regulations, a complementary authorization was obtained on 14-Oct-2019 regarding the ESME research Data Warehouse. All data are exclusively obtained retrospectively, and no procedure is taken to recover unavailable data by contacting healthcare providers or patients.^13^ The present analysis was approved by an independent ethics committee (Comité de Protection des Personnes Sud-Est II-2015-79).

### Objectives

The primary objective of the present study is to assess OS and first-line PFS of HER2+ relapsed MBC based on systemic anti-HER2 adjuvant treatment received, in the whole ESME-MBC cohort. Secondary objective was to identify prognostic factors for OS in this whole cohort and among subgroups.

### Definitions

Standard guidelines are applied to any analysis performed with the ESME data platform. For the ESME-MBC cohort, HER2 and hormone receptor (HR) status were derived from existing results about metastatic tissue sampling when available, or, if not available, from last sampling on early disease. Tumors were defined as HR positive (HR+) if estrogen receptor or progesterone receptor expression was superior or equal to 10% (immunohistochemistry). HER2 immunohistochemical (IHC) score 3+ or IHC score 2+ with a positive fluorescence in situ hybridization (FISH) or chromogenic in situ hybridization (CISH) classified the cancer as HER2+. On the other hand, all cancers with an IHC score 0, 1+, or 2+ with a negative FISH/CISH test, as well as patients with a negative FISH/CISH test without IHC information, were considered as HER2 negative (HER2−). Cancers with an IHC score 2+ without FISH/CISH test information were considered as HER2 unspecified.

### Statistical Analyses

The primary endpoint was OS defined as the time between the date of diagnosis of metastatic disease and date of death for any cause or censored to the date of latest news. PFS under first-line treatment was defined as time between the starting date of first-line treatment and date of first disease progression, or death from any cause, whichever occurs first. Disease-free interval (DFI) was defined as time between date of diagnosis of primary breast cancer and date of first metastasis diagnosis, or death from any cause, whichever occurs first.

Qualitative variables have been described using frequency and percentage distributions. Quantitative data have been described using the number of observations, median, first and thirdquartiles values.

The Kaplan-Meier method was used to estimate survival endpoints (OS and PFS), with the log-rank test to assess the difference between predefined subgroups. The hazard ratio (HR) and associated 95% confidence interval (95%CI) were calculated using a Cox proportional-hazards model. Multivariable Cox proportional hazards models were constructed using a backward step-by-step manual selection procedure to identify time-independent prognostic factors of OS in the whole cohort and for each subtype. All factors significant at a conservative 10% level in univariate analysis were included in multivariable analysis. The final model was reached when including only significant factors at a *P* = .05 significance level. All analyses were carried out using R software (version 3.3.2). sensitivity analyses were conducted among patients with early (6 to 24 months from initial diagnosis) or late relapse (>48 months from initial diagnosis).

## Results

### Study Population Features

**Figure 1.**
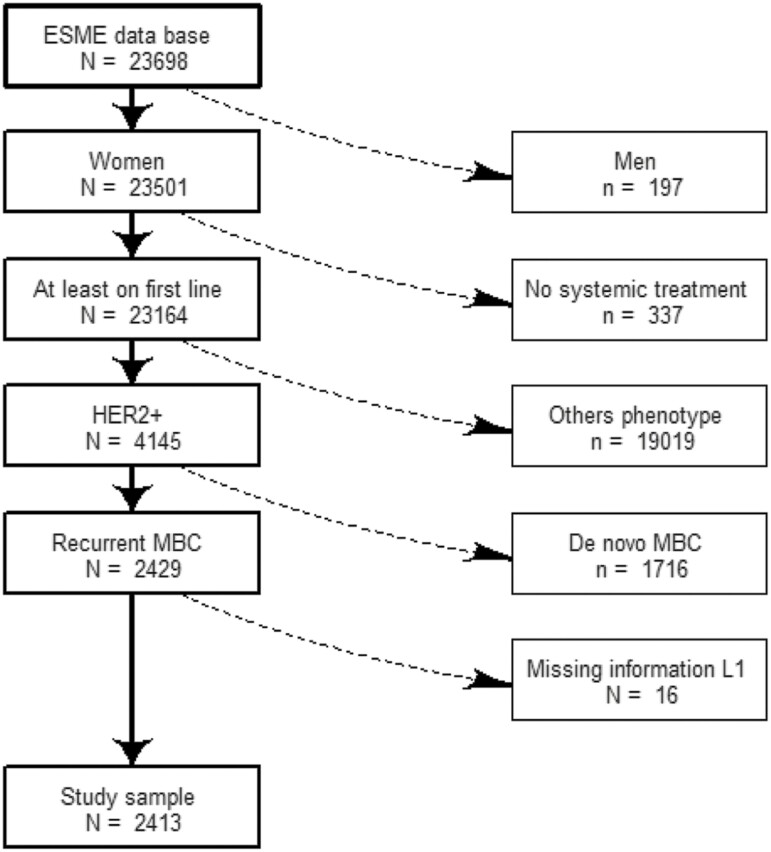
Study flowchart.

From January 2008 and December 2017, the ESME program enrolled 23 698 patients of whom 4145 had an HER2+ MBC. Two thousand four hundred and thirteen (59%) of them had a relapsed MBC, after exclusion of 16 patients with recurrent disease due to missing information on first-line treatment (Fig. 1).

Table 1 summarizes the main demographic and disease characteristics in our population of HER2+ relapsed MBC by previous adjuvant anti HER2 exposure. Among 2413 relapsed HER2+ patients with MBC, 69% had hormone-positive disease. At metastatic diagnosis, 55.5%, 23.6% and 20.9% had respectively 1, 2, or > 2 metastatic sites, respectively. 56% (1343 patients) had received adjuvant trastuzumab and 58% (1392 patients) any adjuvant anti-HER2 targeted treatment, including 2% of other anti-HER2 therapies. Patients pre-exposed to trastuzumab were younger, had a lower disease-free interval, more HR-negative disease and more metastatic sites. Patients pre-exposed to trastuzumab also more frequently presented brain metastases at MBC diagnosis (21,1% versus 6.6% if never exposed).

**Table 1.** Study population and treatments features by pre-exposition to neo/adjuvant anti HER2 treatments among patients with recurrent MBC.

Factor	Pre-exposition status			Total	Chi-square test, *P*-value
	Class	Relapsed not pre-exposed to trastuzumab *N* = 1070	Relapsed and pre-exposed to trastuzumab *N* = 1343		
Period of MBC diagnosis	2008-2012	651	687	1338	<.001
		60.8%	51.2%	55.4%	
	2012-2018	419	656	1075	
		39.2%	48.8%	44.6%	
Age at MBC	18-49	207	520	727	<.001
		19.3%	38.7%	30.1%	
	50-69	525	651	1176	
		49.1%	48.5%	48.7%	
	70 and +	338	172	510	
		31.6%	12.8%	21.2%	
Performance Status at MBC diagnosis	0	236	295	531	.47
		22.1%	22%	22%	
	1	224	269	493	
		20.9%	20%	20.4%	
	2	73	78	151	
		6.8%	5.8%	6.3%	
	3-4	42	41	83	
		3.9%	3.1%	3.4%	
	PS NA	495	660	1155	
		46.3%	49.1%	47.9%	
Histological type	Invasive Ductal Carcinoma (IDC)	770	1146	1916	<.001
		72.9%	85.5%	79.9%	
	Invasive Lobular Carcinoma (ILC)	84	61	145	
		7.9%	4.6%	6,1%	
	IDC and ILC	22	12	34	
		2.1%	0.9%	1.4%	
	Other	181	121	302	
		17.1%	9%	12.6%	
Hormone receptors status	HR−	245	501	746	<.001
		22.9%	37.3%	30.9%	
	HR+	825	842	1667	
		77.1%	62.7%	69.1%	
Number of metastatic sites	1	567	772	1339	.15
		53%	57.4%	55.6%	
	2	271	299	570	
		25.3%	22.3%	23.6%	
	3	140	158	298	
		13.1%	11.8%	12.3%	
	4 and more	92	114	206	
		8.6%	8.5%	8.5%	
Type of metastases	Visceral metastasis	577	617	1194	<.001
		53.9%	45.9%	49.5%	
	Bone only metastatis	178	188	366	
		16.6%	14%	15.2%	
	Brain metastasis	71	284	355	
		6.6%	21.1%	14.7%	
	Other	244	254	498	
		22.9%	19%	20.6%	
Disease-free interval^*^ (months)	6-24	133	400	533	<.001
		12.4%	29.8%	22.1%	
	24-36	95	316	411	
		8.9%	23.5%	17%	
	36-48	87	210	297	
		8.1%	15.6%	12.3%	
	>48	755	417	1172	
		70.6%	31.1%	48.6%	
Systemic treatment received in early setting					
Anthracyclines	No	574	253	827	<.001
		53.6%	18.8%	34.3%	
	Yes	496	1090	1586	
		46.4%	81.2%	65.7%	
Taxanes	No	815	110	925	<.001
		76.2%	8.2%	38.3%	
	Yes	255	1233	1488	
		23.8%	91.8%	61.7%	
Other anti-HER2	No	1065	1299	2364	<.001
		99.5%	96.7%	98%	
	Yes	5	44	49	
		0.5%	3.3%	2%	
Systemic treatment in 1st line metastatic setting					
Type of systemic treatment^*^	HER2-double blockade + taxane	157	261	418	<.001
		17.9%	22.1%	20.3%	
	HER2-double blockade + other	27	48	75	
		3.1%	4.1%	3.6%	
	TDM1	16	72	88	
		1.8%	6.1%	4.3%	
	At least 1 anti-HER2	505	674	1179	
		57.7%	57%	57.3%	
	No anti-HER2	170	127	297	
		19.4%	10.7%	14.4%	
	NA	195	161	356	

^*^From diagnosis of the primary tumor.

Abbreviation: HR: hormone receptor status.

As part of 1^st^-line therapy for MBC, 86% of patients received HER2-targeted agents (74% treated with trastuzumab-based and 24% with trastuzumab-pertuzumab-based regimens) (Table 1).

### Overall Survival

With a median follow-up of 60.7 months (95% CI 58.7-62.3), the median OS of the population of patients with a relapsed HER2+ MBC was 42.8 months (95% CI: 40.7-45.4).

**Figure 2.**
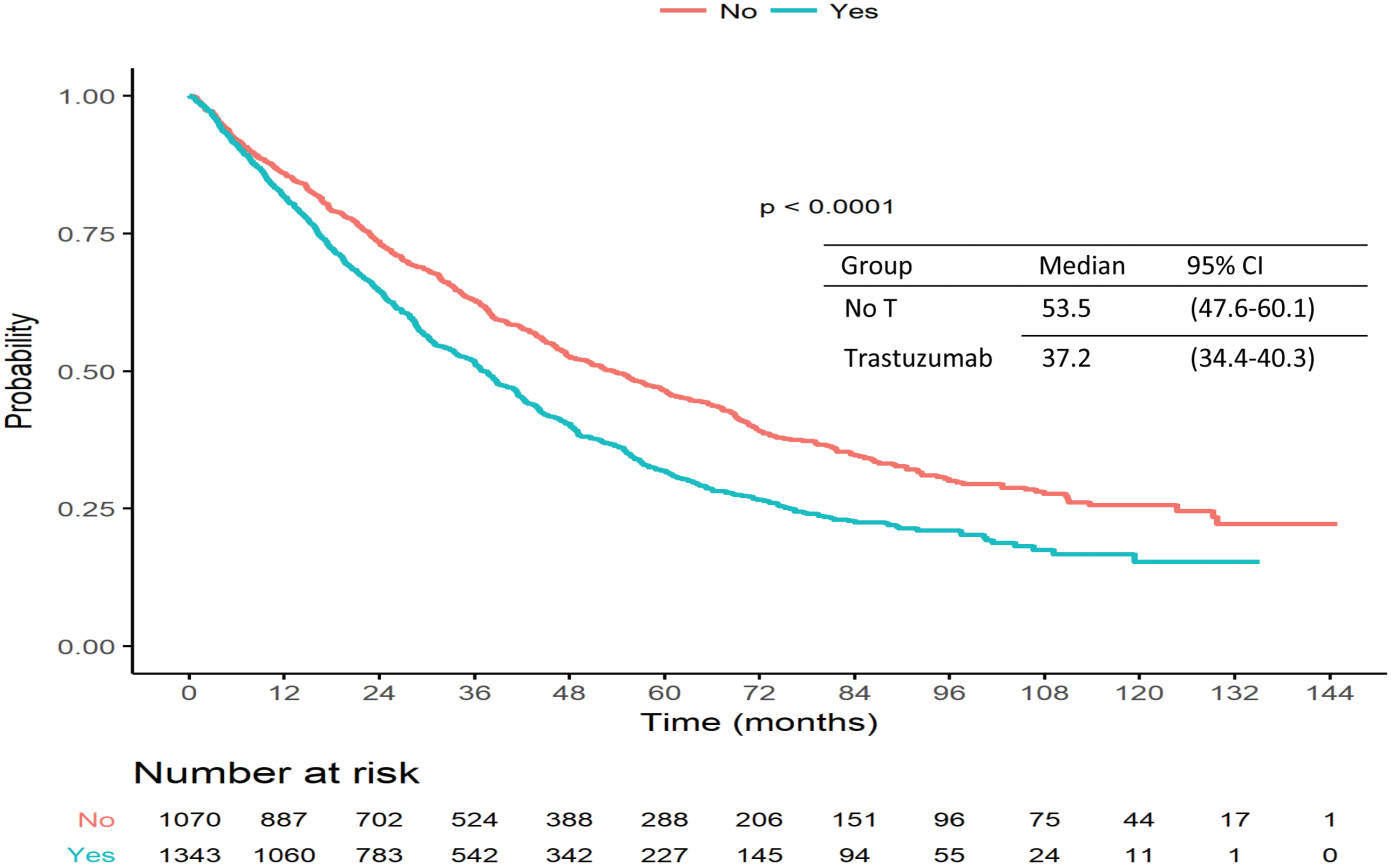
Crude non-adjusted OS in the relapsed HER2+ MBC patients’ cohort according to previous exposure to (neo)adjuvant anti HER2 treatments.

The crude, non-adjusted median OS was inferior in patients who were exposed to adjuvant trastuzumab, as compared to those who did not: 37.2 months (95% CI: 34.4-40.3) versus. 53.5 months (95% CI: 47.6-60.1) (Fig. 2). However, this difference did not persist (HR = 1.07, 95% CI: 0.93-1.24) after adjustment for age, performance status, hormone receptor status, disease-free interval, and number and type of metastatic sites in the multivariable model (Table 2).

**Table 2.** Multivariable Cox model of OS among patients with relapsed HER2+ MBC.

Factors		*N*	HR	IC
Age at MBC	18-49	727	1	
	50-69	1176	**1.23**	**[1.09; 1.4]**
	70 and +	510	**1.74**	**[1.49; 2.05]**
Disease-free interval (months)	6-24	533	1	
	24-36	411	0.86	[0.73; 1]
	36-48	297	**0.63**	**[0.52; 0.75]**
	>48	1172	**0.47**	**[0.41; 0.55]**
Performance status (PS)	PS O	531	1	
	PS 1	493	**1.38**	**[1.15; 1.65]**
	PS 2	151	**2.37**	**[1.86; 3.03]**
	PS 3-4	83	**3.82**	**[2.86; 5.1]**
	PS NA	1155	**2.13**	**[1.84; 2.47]**
Number of metastatic sites	1	1339	1	
	2	570	**1.44**	**[1.25; 1.66]**
	3	298	**1.81**	**[1.52; 2.15]**
	4 and more	206	**2.47**	**[2.04; 2.99]**
Type of metastases	Other visceral	1194	1	
	Bone only	366	**0.79**	**[0.65; 0.95]**
	At least brain	355	**1.23**	**[1.06; 1.44]**
	Other Not visceral	498	**0.72**	**[0.61; 0.84]**
Tumor grade of primary cancer	Grade I	137	1	
	Grade II	903	1	[0.77; 1.29]
	Grade III	917	1.14	[0.88; 1.48]
	Undetermined	456	1.22	[0.93; 1.6]
Hormone receptor (HR) status	HR-	746	1	
	HR+	1667	0.89	[0.79; 1]
Neo(adjuvant) treatment	Chemotherapy only	542	1	
	Chemotherapy + anti-HER2	1322	**1.07**	**[0.93; 1.24]**
	Others ^*^	549	**0.8**	**[0.68; 0.95]**

Bold values indicate *P*-value = 0.

^*^No systemic treatment or endocrine therapy only. Bold values indicate *P*-value = 0.

Abbreviations: NA: not assessed; HR−: hormone receptor negative; HR+: hormone receptor positive.

This was also true in the multivariable analyses conducted in the HR+ and HR− subcohorts, with hazard ratios of 1.07 (95% CI: 0.90-1.27) and 1.14 (95% CI: 0.85-1.52), respectively.

In the global population, a short DFI (6-24 months) remains a strong adverse prognostic factor compared to a later relapse (HR = 2.1, 95% CI: 1.82-2.50 for relapse > 48 months).

### Effect of Adjuvant Trastuzumab Treatment on OS of Recurrent MBC According to Disease-Free Interval

**Figure 3.**
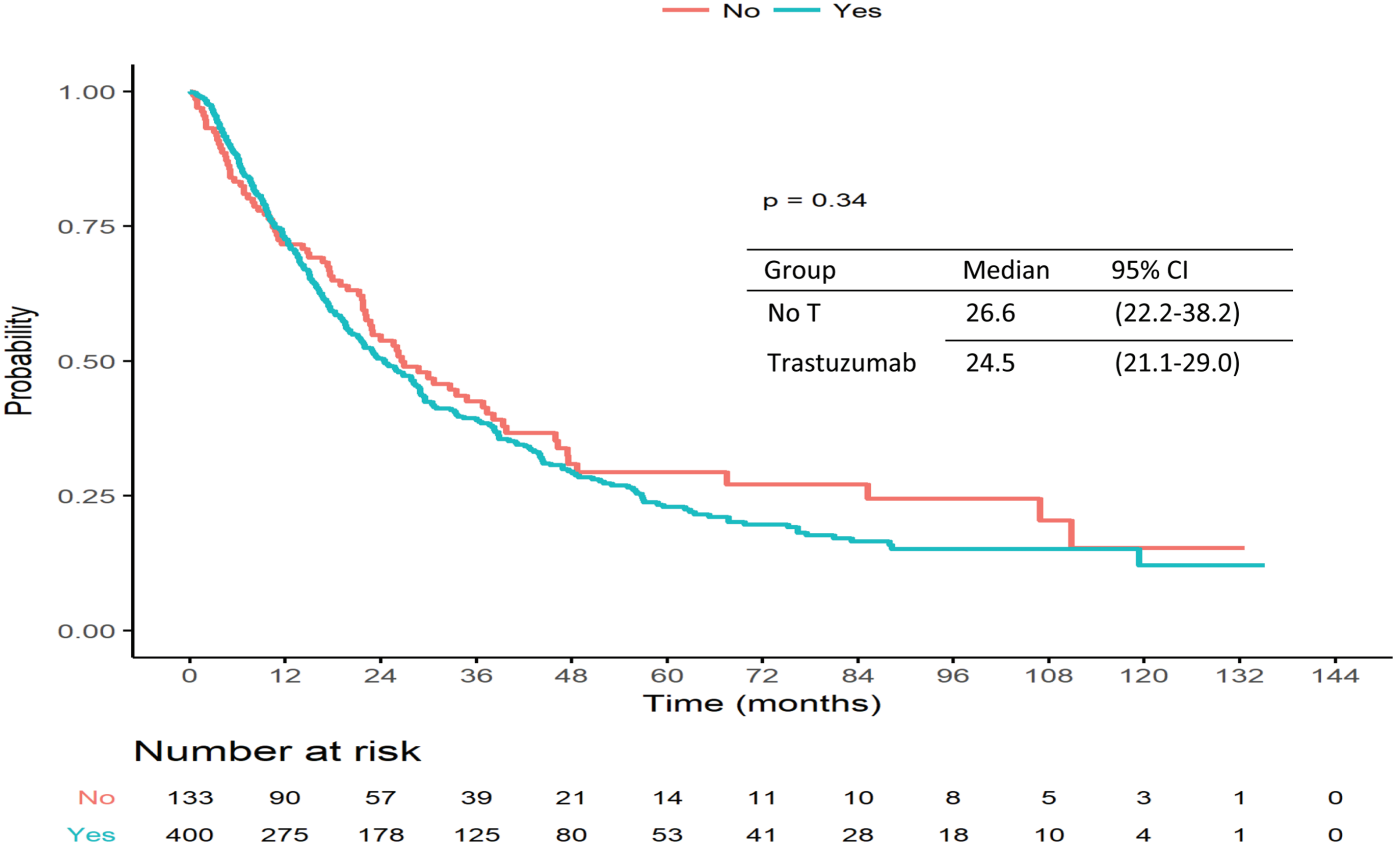
Crude non-adjusted OS in the early relapsed HER2+ MBC patients’ cohort (6-24 months) according to previous exposure to (neo)adjuvant anti HER2 treatments.

For patients with an early relapse (6-24 months), whatever the first-line metastatic treatment, the median OS was not different according to pretreatment with trastuzumab in early setting. The median OS of such patients who received a dual HER2 blockade in the first-line metastatic setting was 28.2 months (19.4-NA) (Fig. 3).

This effect was retained in the subgroup of patients with a late relapse (>48 months from initial diagnosis), which non-adjusted median OS was inferior for patients previously treated by trastuzumab in the early setting, compared to trastuzumab-naïve patients: 49 months (95% CI: 44.8-58) versus 63.1 (95% CI: 56.3-69.4), respectively (HR = 1.27, 95% CI: 1.07-1.5), and persisted in the multivariable model (HR = 1.30, 95% CI: 1.07-1.58) (Supplementary Fig. 1A). This effect seemed less pronounced in patients who received a dual blockade as first-line treatment (single HER2 blockade (*N* = 525 patients), HR = 1.78, 95% CI: 1.41-2.25, *P* < .0001 for trastuzumab pretreated versus trastuzumab naïve patients, versus dual blockade (*N* = 262 patients), HR = 1.05, 95% CI: 0.65-1.7, *P* = .84) (Supplementary Fig. 1B and C).

### First-Line PFS of Recurrent MBC

**Figure 4.**
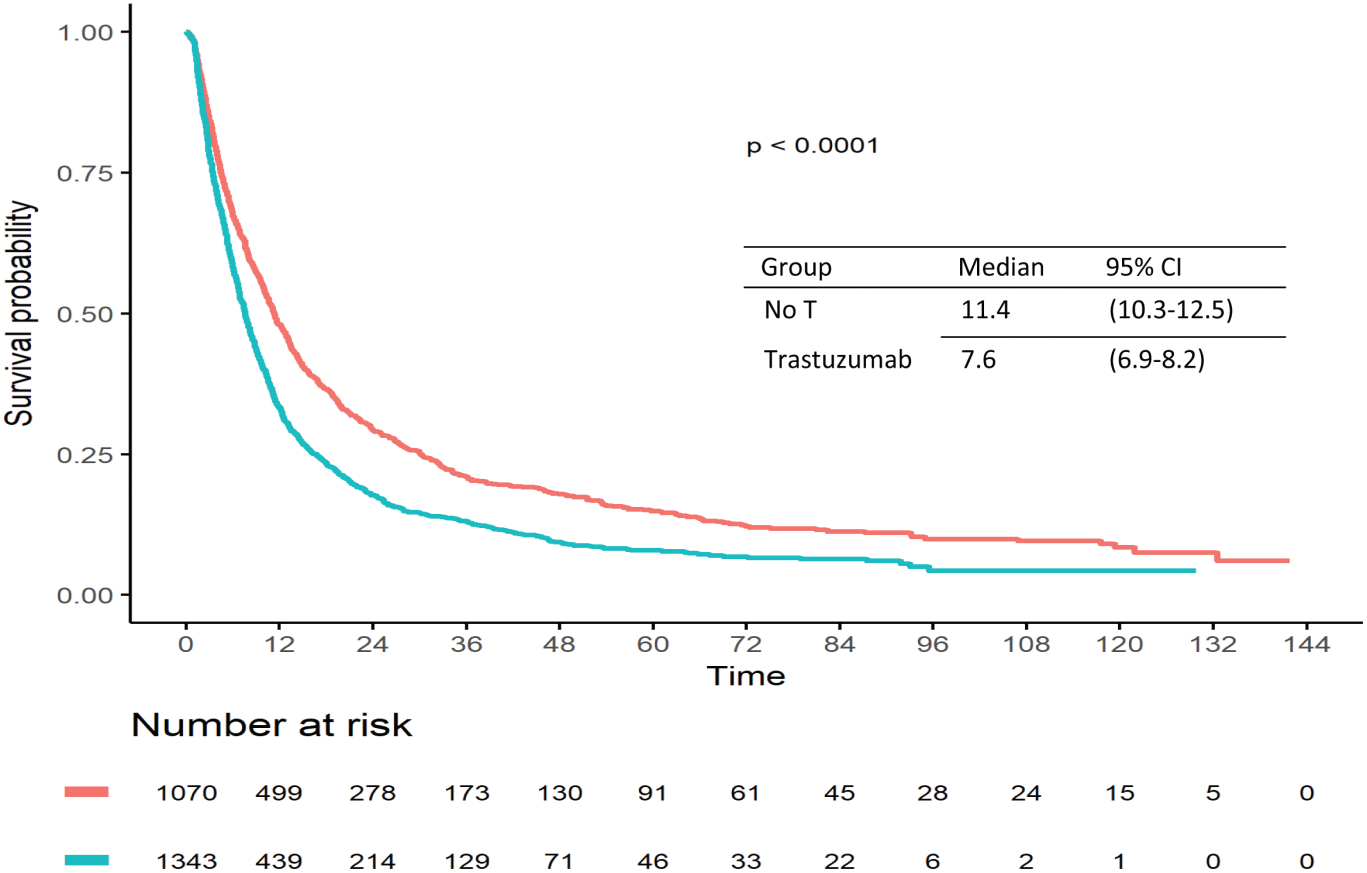
Crude PFS in the relapsed HER2+ MBC patients’ cohort according to previous exposure to (neo)adjuvant anti HER2 treatments.

The non-adjusted median PFS under first-line therapy (PFS1) was inferior in patients who had received neo/adjuvant anti-HER2 treatment, as compared to those who had not: 7.6 months (95% CI: 6.9-8.2) versus. 11.4 months (95% CI: 10.3-12.5) (Fig. 4). However, as for OS, this difference did not persist after adjustment for age, performance status, DFI and number and type of metastatic sites in the multivariable model (HR = 1.05, 95% CI: 0.93-1.18).

## Discussion

In our cohort, anti HER2 pre-exposure in the neo/adjuvant setting is not an independent adverse prognostic factor among patients with relapsed HER2-positive MBC, despite a shorter crude OS and progression-free survival.

Characteristics of our population is also quite consistent with other real-life registries (59% of patients has relapsed MBC versus 50% in SystHERs) or clinical trials in first-line metastatic setting (from 65% in PERUSE trial to 47% in CLEOPATRA trial).^14-16^

However, impact of adjuvant anti-HER2 targeted treatment on outcomes in metastatic setting of HER2+ relapsed patients with breast cancer are still undetermined, even if there is a trend toward a lower benefit in patients recurring after trastuzumab in early setting.^17-21^

In relapsed patients with MBC, we observed a difference in term of median OS between patients previously exposed to trastuzumab and those who were not, with a crude gap of 16.3 months. Further data come from PERUSE trial evaluating dual blockade with pertuzumab, trastuzumab plus paclitaxel in first-line metastatic setting, with a similar trend of a reduced OS for of trastuzumab pre-treated patients of 54.1 months (95% CI: 48.7-60.7) versus 73.5 months (95% CI: 65.6-NE) for trastuzumab-naïve patients.^16^ However, in the ESME-cohort, adjuvant trastuzumab administration does not predict for worse outcomes when adjusted to the other prognostic factors.

For patients with early relapse (6-24 months), whatever anti-HER2 used in first line, median OS is not different between patients previously treated or not by trastuzumab in early setting, suggesting a resistance to anti-HER2-targeted treatment. A short disease-free interval (6-24 months compared to >48) is confirmed to be a strong adverse prognostic factor (HR=2.1, 95% CI: 1.82-2.50), as previously reported.^9,14^

Patients with *late relapse (>48 months)* treated by trastuzumab in early setting, experienced a worse OS than trastuzumab naïve patients, which remains significant in the multivariate model. This difference seems to be driven by patients treated in first line of metastatic setting by trastuzumab as one and only anti-HER2 blockade. Indeed, in this subgroup of patients, a significant difference in term of OS was demonstrated but not for patients with late relapse treated by HER2 dual blockade in first line of metastatic setting. This could suggest, only in this specific population of late relapsed MBC, an ATRESS phenomenon that could be overcome with the use of an additional anti-HER2-driven treatment. In the CLEOPATRA trial, the small subgroup of patients (*n* = 88) previously exposed to trastuzumab in the (neo)adjuvant setting had the same PFS than trastuzumab naïve patients, thus confirming the advantage of adding pertuzumab even in patients progressing after early trastuzumab-based chemotherapy.^22^

A similar trend toward decreased benefit in trastuzumab pre-treated patients was observed for PFS under first line of systemic treatment. However, in multivariable model, administration of adjuvant trastuzumab does not predict for worse outcomes when adjusted to the other prognostic factors. In the SystHERs, patients with relapsed MBC (65.8% were pre-exposed to trastuzumab compared to 55.7% in ESME cohort) had a longer median PFS of 11.9 months (8.9 months in our ESME cohort). Consistently, this is more likely due to a wider use of dual blockade in first line in the SystHERs (59.8% versus 20.3%). However, both data could suggest that the “real-life” benefit from dual blockade in trastuzumab pre-treated patients is smaller than what was demonstrated in CLEOPATRA. In CLEOPATRA, only 10% of patients received adjuvant trastuzumab. However, pre-treated patients (with a minimal trastuzumab free-interval of 12 months) presented a worse PFS compared to trastuzumab naïve patients (16.9 versus 21.6 months). This trend was confirmed in PERUSE and the SUPER trial which tested the CLEOPATRA regimen.^16,22,23^

Interestingly, in our cohort, compared to de novo MBC, relapsed MBC experienced more frequently *brain metastases* at MBC diagnosis (14.7% versus 4.8%), as previously described in ESME cohort.^24^ In the KATHERINE trial, with a median 41.2 months follow-up, use of T-DM1 in adjuvant post-neoadjuvant setting, does not seem to impact incidence of central nervous system recurrence as first site of recurrence (4.3% in the trastuzumab arm versus 5.9% in the T-DM1 arm); however, a longer follow-up remains necessary.^8^ New treatment options are evaluating impact of tucatinib and trastuzumab deruxtecan in early setting (NCT04457596, NCT04622319).^25^

Our study presents some limitations: patients included in this study were not eligible to T-DM1 in adjuvant setting due to enrollment period (2008-2019), as currently recommended. There is a low rate of dual blockade in early setting as pertuzumab in neoadjuvant and adjuvant setting is not reimbursed in France, precluding a robust evaluation of this population. Moreover, use of and addition of endocrine therapy as maintenance during dual blockade in 1st line was not a standard of care (and could not be accurately evaluated in our study).

Changing treatment landscape in MBC with new treatment options (ie, trastuzumab deruxtecan and tucatinib) could be better alternatives to the re-induction of regimens already used in early setting. However, results of ongoing clinical trial of this recent compounds in early stage could lead in a near future to similar questions about re-induction in late stage.

## Conclusion

In our study, the receipt of adjuvant trastuzumab does not predict worse outcomes when adjusted to the other prognostic factors, among patients with HER2+ MBC who relapsed during the 2008-2017 period. However, in our population of interest of relapsed HER2+ BC patients, an ATRESS phenomenon could not be ruled out for patients who experienced late relapse.

Real-world data as ESME cohort add a better knowledge to treatment efficacy when guidelines with new therapies are implemented. The population treated in clinical trials today is far more different than those described in pivotal trials which led to approval as CLEOPATRA.

## Supplementary Material

oyad137_suppl_Supplementary_Material

## Data Availability

The data underlying this article cannot be shared publicly due to French data protection authority. This work was authorized by the French data protection authority ([Registration ID 1704113 and authorization N_DE-2013.-117], NCT03275311). Moreover, in compliance with the applicable European regulations, a complementary authorization was obtained on 14 October 2019 regarding the ESME research Data Warehouse. All data are exclusively obtained retrospectively, and no procedure is taken to recover unavailable data by contacting healthcare providers or patients.^13^ The present analysis was approved by an independent ethics committee (Comité de Protection des Personnes Sud-Est II-2015-79). The data will be shared on reasonable request to the corresponding author.
