# Supplementary material for: Real-World Impact of Adjuvant Anti-HER2 Treatment on Characteristics and Outcomes of Women With HER2-Positive Metastatic Breast Cancer in the ESME Program
Source: Oncologist. 2023 Aug 17;28(10):e867–76. doi: 10.1093/oncolo/oyad137 (PMC10546827; doi:10.1093/oncolo/oyad137)
Supplement: oyad137_suppl_Supplementary_Material [file oyad137_suppl_supplementary_material.docx]

**SUPPLEMENTAL DATA**

**Figure S1:** Crude non-adjusted OS according to previous exposure to (neo)adjuvant anti HER2 treatments: A) in the late relapsed HER2+ MBC patients’ cohort (>48 months); B) in the late relapsed cohort treated with HER2-single blockade in 1^st^ line MBC; C) in the late relapsed cohort treated with HER2-dual blockade in 1^st^ line MBC;


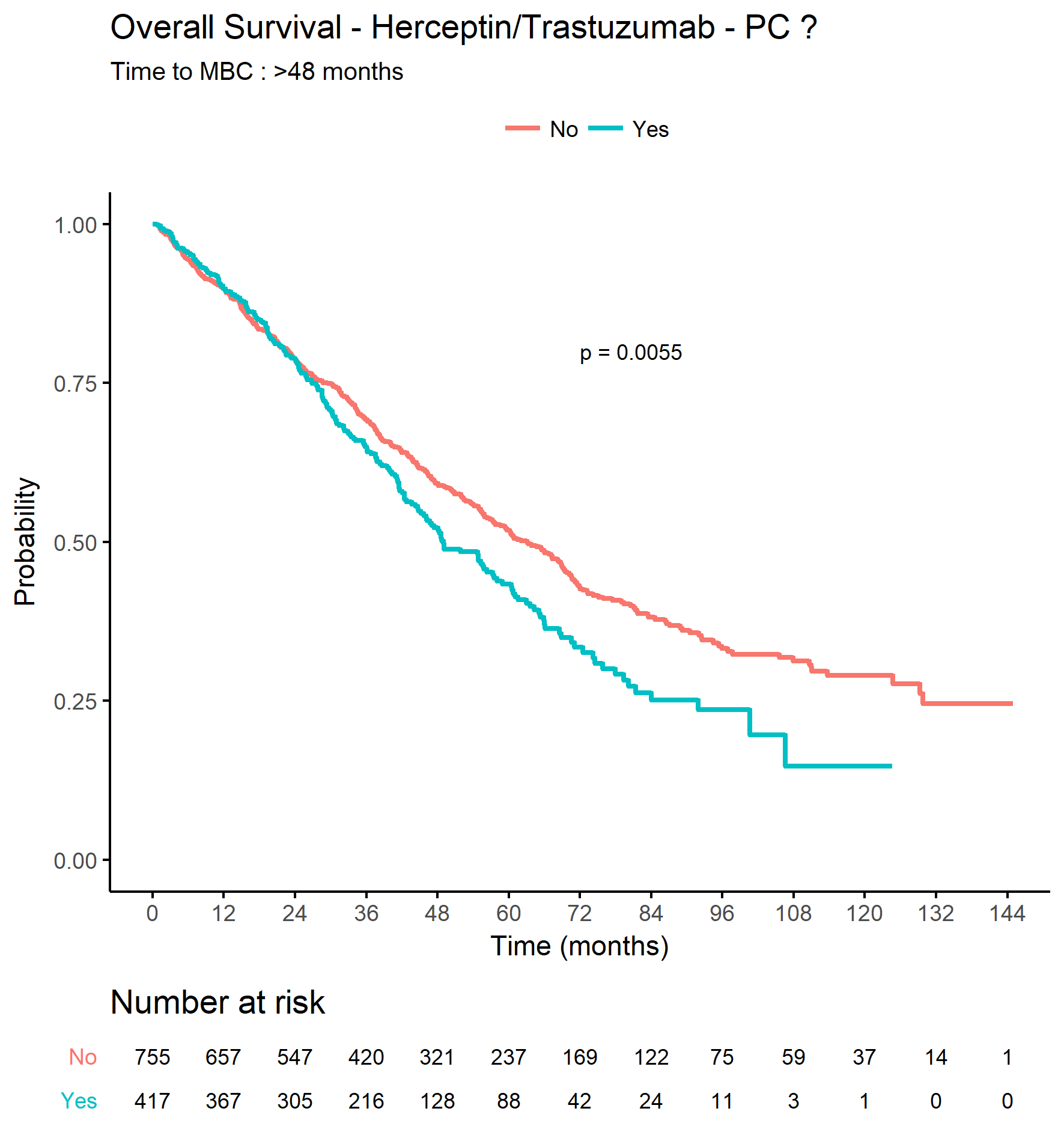


**A.**

| Group | Median | 95% CI |
| --- | --- | --- |
| No T | 63.1 | (56.3-69.4) |
| Trastuzumab | 49.0 | (44.8-58.0) |


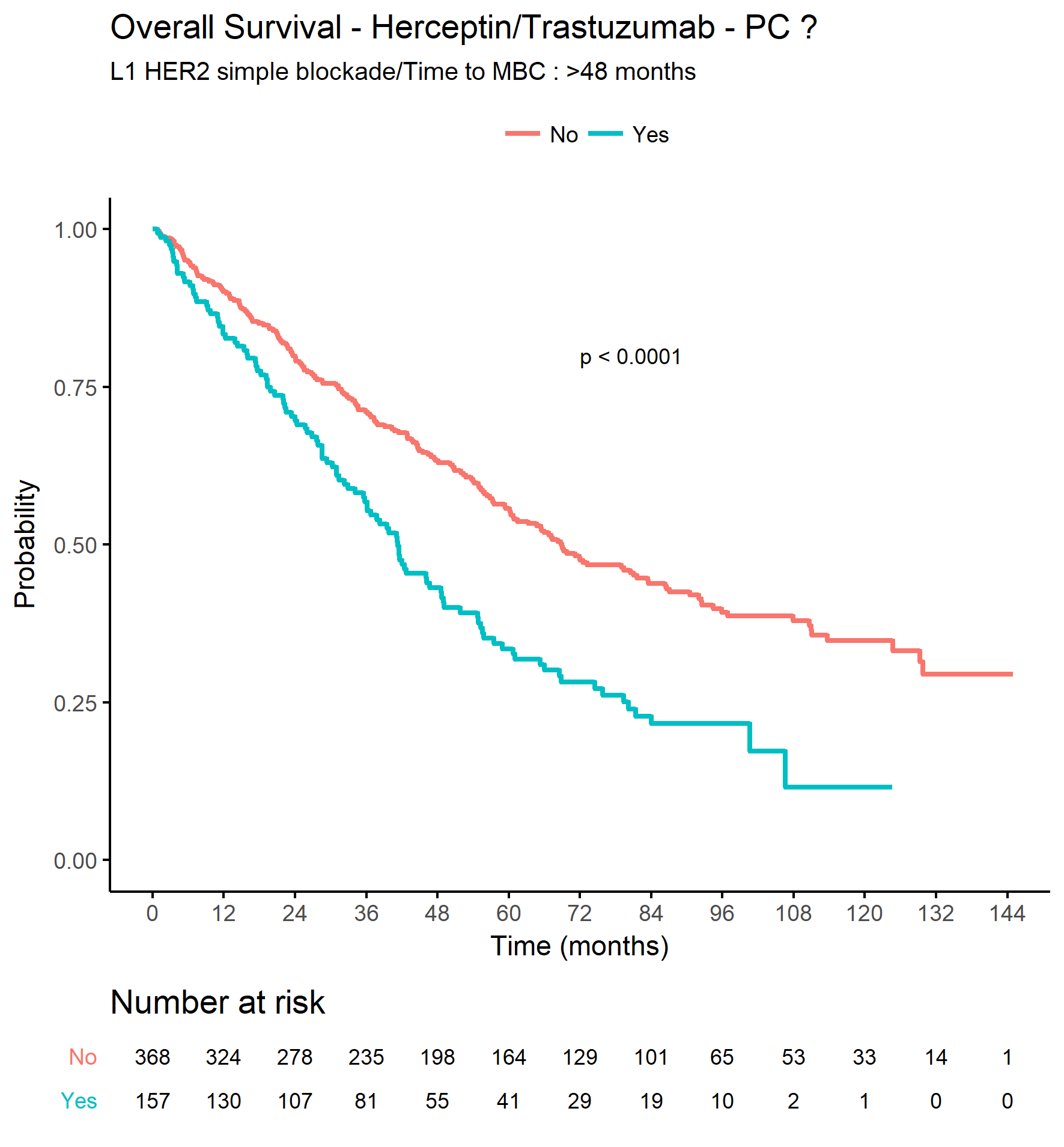


**B.**

| Group | Median | 95% CI |
| --- | --- | --- |
| No T | 68.9 | (60.2-83.5) |
| Trastuzumab | 41.3 | (35.7-49.0) |


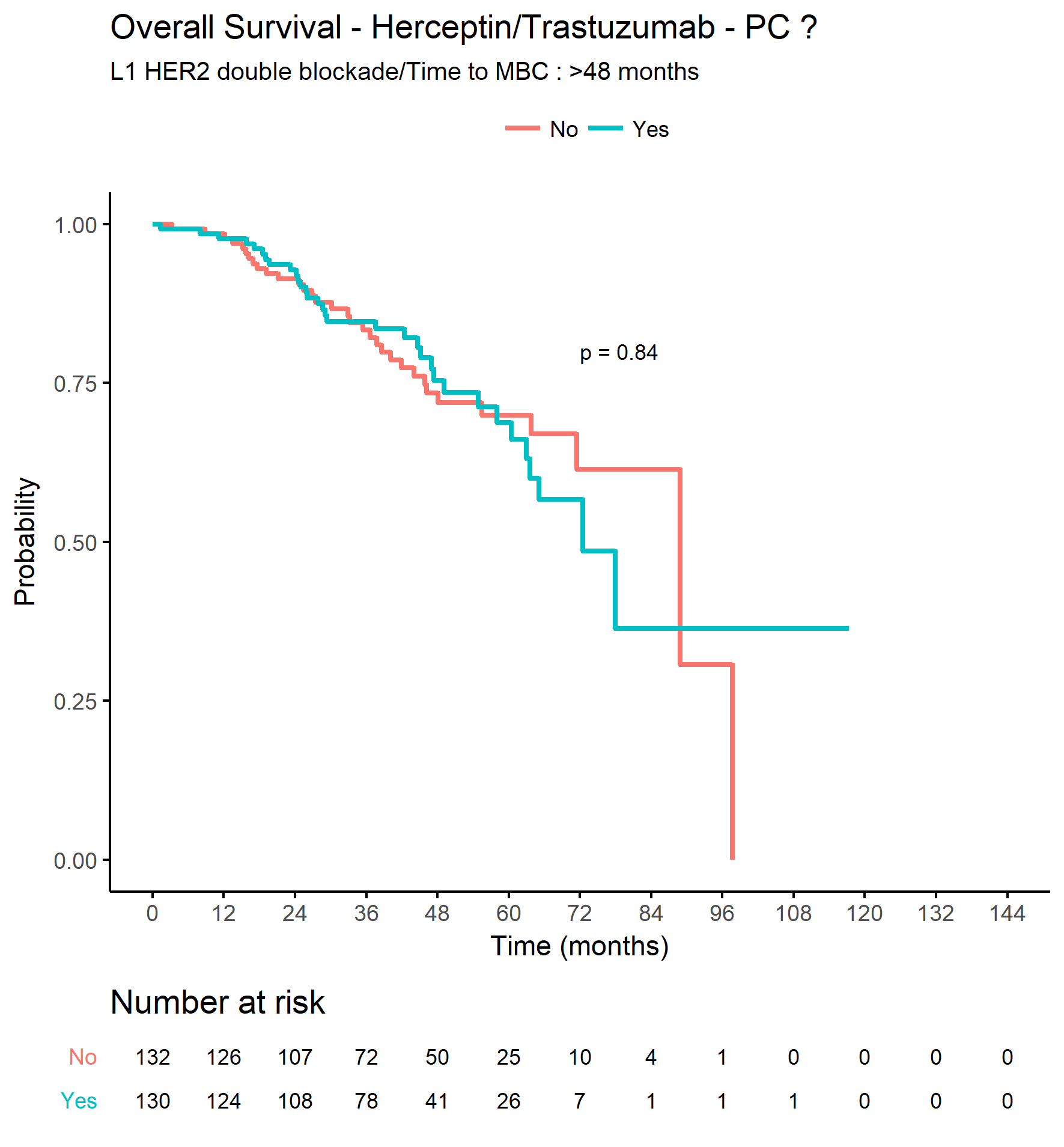


| Group | Median | 95% CI |
| --- | --- | --- |
| No T | 88.9 | (71.5-NA) |
| Trastuzumab | 72.5 | (63.6-NA) |
| Group | Median | 95% CI |
| No T | 88.9 | (71.5-NA) |
| Trastuzumab | 72.5 | (63.6-NA) |

**C.**

Bas du formulaire
